# MR compatible detectors assessment for a 0.35 T MR-linac commissioning

**DOI:** 10.1186/s13014-024-02431-8

**Published:** 2024-03-20

**Authors:** Michel Chea, Mathilde Croisé, Christelle Huet, Céline Bassinet, Mohamed-Amine Benadjaoud, Catherine Jenny

**Affiliations:** 1 Medical Physics Department, Pitié-Salpêtrière Hospital, AP-HP Sorbonne University, 47-83 Boulevard de l’Hôpital, 75651 Paris Cedex 13, France; 2 Institut de Radioprotection et Sûreté Nucléaire (IRSN), PSE-SANTE/SDOS/LDRI, 92260 Fontenay-aux-Roses, France; 3 Institut de Radioprotection et Sûreté Nucléaire (IRSN), PSE-SANTE/SERAMED, 92260 Fontenay-aux-Roses, France

**Keywords:** Commissioning, Small fields, Magnetic field, Detectors, Diodes, Microdiamond, Output factors, MR-linac, Air gap

## Abstract

**Purpose:**

To assess a large panel of MR compatible detectors on the full range of measurements required for a 0.35 T MR-linac commissioning by using a specific statistical method represented as a continuum of comparison with the Monte Carlo (MC) TPS calculations. This study also describes the commissioning tests and the secondary MC dose calculation validation.

**Material and methods:**

Plans were created on the Viewray TPS to generate MC reference data. Absolute dose points, PDD, profiles and output factors were extracted and compared to measurements performed with ten different detectors: PTW 31010, 31021, 31022, Markus 34045 and Exradin A28 MR ionization chambers, SN Edge shielded diode, PTW 60019 microdiamond, PTW 60023 unshielded diode, EBT3 radiochromic films and LiF µcubes. Three commissioning steps consisted in comparison between calculated and measured dose: the beam model validation, the output calibration verification in four different phantoms and the commissioning tests recommended by the IAEA-TECDOC-1583.

**Main results:**

The symmetry for the high resolution detectors was higher than the TPS data of about 1%. The angular responses of the PTW 60023 and the SN Edge were − 6.6 and − 11.9% compared to the PTW 31010 at 60°. The X/Y-left and the Y-right penumbras measured by the high resolution detectors were in good agreement with the TPS values except for the PTW 60023 for large field sizes. For the 0.84 × 0.83 cm^2^ field size, the mean deviation to the TPS of the uncorrected OF was − 1.7 ± 1.6% against − 4.0 ± 0.6% for the corrected OF whereas we found − 4.8 ± 0.8% for passive dosimeters. The mean absolute dose deviations to the TPS in different phantoms were 0 ± 0.4%, − 1.2 ± 0.6% and 0.5 ± 1.1% for the PTW 31010, PTW 31021 and Exradin A28 MR respectively.

**Conclusions:**

The magnetic field effects on the measurements are considerably reduced at low magnetic field. The PTW 31010 ionization chamber can be used with confidence in different phantoms for commissioning and QA tests requiring absolute dose verifications. For relative measurements, the PTW 60019 presented the best agreement for the full range of field size. For the profile assessment, shielded diodes had a behaviour similar to the PTW 60019 and 60023 while the ionization chambers were the most suitable detectors for the symmetry. The output correction factors published by the IAEA TRS 483 seem to be applicable at low magnetic field pending the publication of new MR specific values.

## Introduction

MR guided radiotherapy (MRgRT) represents a new paradigm for day to day adaptive treatments indicated for “hard to treat” tumors. The revolutionary combination of an MRI system within a linear accelerator provides better contrast images than kV X-rays or cone beam computed tomography, especially for soft tissues. This is a major asset for both adaptive planning session with improvement of the patient treatment based on daily anatomy and radiation-free real time imaging through the entire treatment fraction.

The two main commercially available MR-linacs are the Unity (Elekta, Sweden) and the MRIdian (Viewray, Oakwood Village, OH, USA) with a 1.5 T and a 0.35 T magnetic field respectively. For the latter, the magnetic field is oriented towards the bore, from the patient’s feet to the head and perpendicular to a 6 MV FFF beam. The beam is defined by a double focused and stacked MLC of 138 leaves. The upper and the lower MLC are shifted by half a leaf to have a width leaf of 0.415 cm at SAD 90 cm and to minimize interleaf leakage [1]. The minimum and the maximum field size available are 0.2 × 0.415 cm^2^ and 27.4 × 24.07 cm^2^ respectively. MRIdian TPS uses an optimized Monte Carlo code based on VMC with variance reduction techniques implemented to reduce computation time for routine clinical use [2]. MRIdian system comes with a complete beam model from the manufacturer. During the installation, the MRIdian is tuned to match the Viewray references based on the golden beam data inserted in the TPS. Then the system acceptance tests (SAT) performed with both Viewray and the site physicists consist in ensuring that the linac is consistent with the Viewray set of golden beam data through specific measurements. The detectors recommended by the vendors are:

- The Exradin A28 MR in a 1D water tank to measure the output calibration and three percentage depth doses (PDD) (3.32 × 3.32 cm^2^, 9.96 × 9.96 cm^2^ and 27.2 × 24.07 cm^2^).
- A Sun Nuclear (SN) Edge for the output factors measurements
- A SN Edge and an IC Profiler (Sun Nuclear, Melbourne, FL, USA) for profiles checking respectively for small and large fields.

To enable the linear accelerator to operate correctly in the presence of the static magnetic field, it is isolated from the MR system thanks to six shielding compartments mounted upon the gantry. However, the secondary electrons deviation dropped off in the medium cannot be prevented, leading to a shifted and distorted dose distribution. The dose kernel becomes asymmetric making the percentage depth dose (PDD) shifted towards the surface [3]. For a 1.5 T magnetic field, the central axis offset can vary from 1 to 1.7 mm depending on the field size and depth; the penumbra perpendicular to the magnetic field can be asymmetric by 1 mm [4]. In presence of heterogeneities, an electron return effect increases the dose at tissue air boundaries and the electrons have an arc-shaped trajectory in air cavities. But these effects are reduced at lower magnetic field strength [5].

The magnetic field leads to measurement issues due to the Lorentz force. Due to the unavoidable necessity to use phantoms throughout the quality assurance (QA) process, the ESTRO-ACROP guideline recommends evaluating the dosimetric effect of air gaps around the detector for phantom measurements [6]. Hackett et al. advise to perform reference dosimetry measurements in water only. Under a 1.5 T magnetic field, the collected charges are reduced from 0.7 to 1.2% in a plastic phantom and a large angular dependence is observed, attesting the impact of air distribution around the chamber [7]. In their Monte Carlo study performed with a PTW 30013 Farmer chamber under a 1.5 T magnetic field, Obrien et al. modelled various symmetric and asymmetric air gap widths and found a small effect for symmetric air gaps, inferior to 0.5% for a 1.4 mm thickness, compared to asymmetric air gaps, up to 1.6% for a 0.2 mm thickness [8]. Finally, Margaroni et al. investigated twelve ionization chambers dose response under a 1.5 T magnetic field with MC simulations: the asymmetrical air gap effect for small-cavity ionization chambers was considerably higher than for Farmer type ionization chambers and still significant even for a 0.1 mm air gap. It was observed that the detector orientation parallel to the magnetic field reduced the air gap effect and showed negligible dose response variations according to the field size, depth and material [9].

For reference dosimetry measurements, a beam quality correction factor has been recently introduced to determine the absorbed dose to water under magnetic field. The usual k_Q,Q0_ described in the IAEA TRS-398 is replaced by k_B_,_Q,Q0_. Several k_B_,_Q,Q0_ mainly for Farmer type chambers have been published for two different chamber orientations. Deviations up to 4.2% are reported between k_B_,_Q,Q0_ parallel and perpendicular to the magnetic field [3, 10–12]. Farmer type chambers are usually used for reference dose output calibration but definitely not suitable for commissioning end to end tests. Only Krauss et al. [10] proposed k_B_,_Q,Q0_ factors for recently released detectors down to 0.015 cc.

Concerning relative measurements under a 1.5 T magnetic field, the shielded diodes inhibit the effect of the Lorentz force making the profile more symmetrical than it actually is and the unshielded diodes over-respond out of the field. Generally, diodes are not recommended for output factors (OF) measurements in the presence of a magnetic field [13]. The diode non-water equivalent density components have been identified as the major contributors to their behaviour in magnetic field and a strong dependence of the small field output correction factors with the magnetic field strength has been found [14]. Woodings et al. compared the PTW 60019 microdiamond detector to a small ionization chamber and they considered it suitable for small field measurements and MR-linac commissioning. Nevertheless, they found an important angular response at 1.5 T magnetic field intensity making this detector not appropriate for characterising the profiles with parameters such as symmetry [15]. The PDD and the profiles measured by Chen et al. with a micro-ionization chamber (sensitive volume of 0.015 cc) and a microdiamond matched the TPS beam model for field size from 1 × 1 cm^2^ to 10 × 10 cm^2^ but with a sharper penumbra for the microdiamond [16]. Lim et al. compared output factors and profiles measured with EBT-XD films against PTW diamond detector, Exradin A26 microchamber and Monte Carlo TPS calculation for small field size down to 1 × 1 cm^2^: if used carefully with a specific protocol, EBT-XD films can provide accurate dosimetric results and can take into account the electrons return effect [17].

For relative measurements under a 0.35 T magnetic field, the literature is rather poor. Two studies [18, 19] found a good agreement between measured output factors (Sun Nuclear Edge, PTW 60019 and PTW 31021) and TPS output factors down to a 1.7 × 1.7 cm^2^ field size.

While many articles about the Unity commissioning are available [4, 16, 20–23], only one paper about the MRIdian has been published yet [19]. However, these studies on the behaviour of detectors under magnetic field have focused on few detectors and on field sizes that don’t cover the full range, especially small field sizes, required for the commissioning. In addition, the impact of a low magnetic field, such as the 0.35 T available on the MRIdian, needs to be better investigated. Indeed, the significant difference between parallel and perpendicular k_B_,_Q,Q0_ for 0,35 T attests to the non-negligible effect of a low magnetic field on measurements [10].

First, this study aims at evaluating a large panel of MR-compatible waterproof detectors for PDD, profiles, and output factors measurements required for commissioning a 0.35 T MR-linac including very small field sizes. The assessment was performed using a specific statistical method innovatively represented as a continuum of comparison with the Monte Carlo TPS calculations. In addition, the impact of air gap and material on the absolute dose measurement has been investigated. Finally, this study describes MRIdian commissioning tests and the secondary Monte Carlo dose calculation (Zeus) validation.

## Material and methods

The commissioning that consisted in comparison between calculated and measured dose was carried out in three steps: the beam model validation, the beam output calibration verification and the commissioning tests. Through these steps, PDD, profiles, output factors and absolute dose points were extracted from the TPS and compared to measurements performed with ten different MR compatible detectors whose characteristics and use in this study are reported in Table 1.

**Table 1:** Characteristics of the detectors and their use in the study

	Detectors	Collecting volume	Constructor field size range of use	Type of measurement performed
Ionization chambers	Exradin A28 MR	0.125 cc	3–40 cm^2^	Output calibration
	PTW Semiflex 31010	0.125 cc	3–40 cm^2^	Output calibration/PDD/Profiles/Output factors
	PTW Semiflex 3D 31021	0.07 cc	2.5–4 cm^2^	Output calibration/PDD/Profiles/Output factors
	PTW Markus 34045	0.02 cc	3–40 cm^2^	PDD
	PTW Pinpoint 3D 31022	0.016 cc	0.8–40 cm^2^	PDD/Profiles/Output factors
High resolution detectors	Sun Nuclear Edge (Shielded diode)	0.019 mm^3^	0.5–10 cm^2^	PDD/Profiles/Output factors
	PTW 60019 microDiamond	0.004 mm^3^	0.4–40 cm^2^	PDD/Profiles/Output factors
	PTW 60023 microSilicon (unshielded diode)	0.03 mm^3^	0.4–10 cm^2^	PDD/Profiles/Output factors
Passive detectors	EBT3 radiochromic films	N/A	N/A	Output factors
	Lif microcubes	1 mm^3^	N/A	Output factors

### Reference data: TPS calculation

Plans were created on the Viewray Treatment Planning Station (Version 5.4.0.97) to generate Monte Carlo reference data under 0.35 T magnetic field with 1 mm resolution and 0.5% accuracy. The relative reference data were calculated on a numerical water phantom with a voxel size of 1 × 1 × 1 mm^3^ and the investigated field sizes varied from 0.42 × 0.415 cm^2^ to 27.2 × 24.07 cm^2^. PDD and profiles were extracted. The TPS profiles were fitted with a bivariate penalized spline function and the resulting curves were resampled with a 0.1 mm resolution for a better accuracy. For the output factors, output calibration verification and the commissioning tests, calculated absolute dose points were collected.

### Relative dose measurements for beam model validation

All the measurements with active detectors were performed with the Beamscan MR water tank (PTW, Freiburg, Germany). The studied field sizes ranged from 0.42 × 0.415 cm^2^ to 27.2 × 24.07 cm^2^.

The PDD were measured at 78 cm SSD with seven active detectors (PTW 34045 Markus, PTW 31010, PTW 31021 and PTW 31022 ionization chambers, PTW 60019 microdiamond, PTW 60023 and SN Edge diodes).

The X/Y profiles were measured at 85 cm SSD and 5 cm depth with six active detectors (PTW 31010, PTW 31021, PTW 31022, PTW 60019, PTW 60023 and Sun Nuclear Edge). For the comparison between measured and calculated profiles, the following usual parameters were analysed: field size, penumbra [24], unflatness and symmetry [25] (“Appendix A”).

**Fig. 1:**
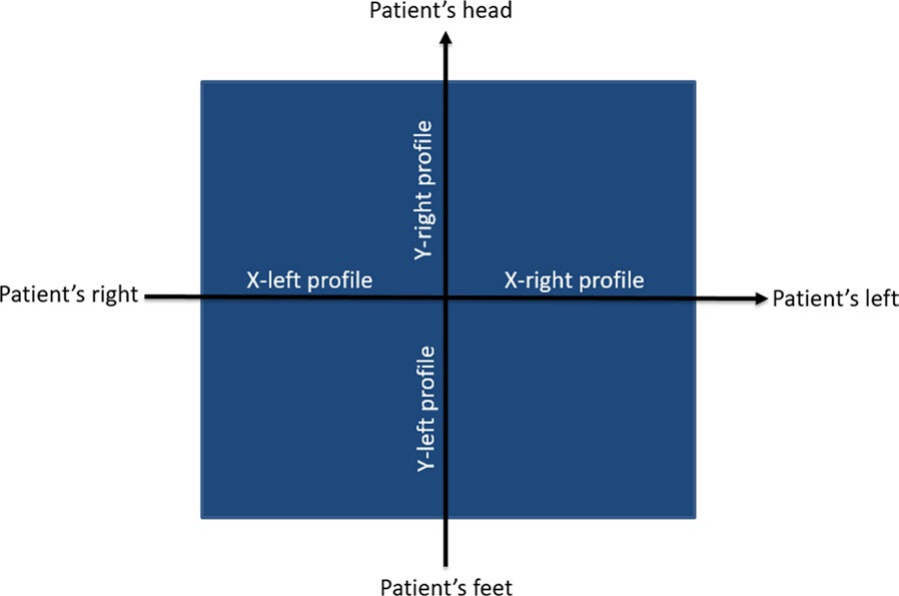
Profiles orientation. View of the field from the gantry at 0° with the patient in head first supine position

Figure 1 represents the profiles orientation in a view from the gantry at 0° when the patient is in head first supine position. The X-profiles and the Y-profiles are in the patient’s right to left and feet to head direction respectively.

For a better interpretation of the profile results some complementary measurements were performed. Firstly, additional EBT3 film profiles for the 0.84 × 0.83 cm^2^ field size were carried out. Secondly, angular response of high resolution detectors relative to the PTW 31010 ionization chamber was assessed in the water tank (to avoid air gap effects), at SSD 78 cm, 12 cm depth with a 4.16 × 4.15 cm^2^ field size. The PTW 60019 microdiamond and PTW 60023 diode were oriented parallel to the beam at gantry 0° (perpendicular to the magnetic field direction) while the PTW 31010 ionization chamber and the SN Edge diode were perpendicular to the beam. Due to the limited space of the water tank in the MR-linac, angles from − 60 to + 60° with 10° steps were evaluated. To highlight the magnetic field impact, the same measurements were performed on a Truebeam Stx (Varian, Palo Alto, CA, USA) with a 6 MV FFF beam of 4 × 4 cm^2^ field size. Due to the water tank dimensions, the experimental set-up was at SSD 97 cm and 3 cm depth.

Output factors were measured at 85 cm SSD and 5 cm depth with six active detectors (PTW 31010, PTW 31021, PTW 31022, PTW 60019, PTW 60023 and Sun Nuclear Edge) and two passive dosemeters (LiF µcubes and EBT3 radiochromics films). Output factors with active detectors were performed from field sizes ranging from 0.42 × 0.415 cm^2^ to 27.4 × 24.07 cm^2^ whereas passive dosemeters were used for small field measurements, i.e. from 0.42 × 0.415 cm^2^ to 9.96 × 9.96 cm^2^; the reference field being 9.96 × 9.96 cm^2^ in both cases. OF measurements with the passive dosemeters were performed in a virtual water phantom. For EBT3 films, a calibration curve from 0.5 to 4 Gy was performed at 85 cm SSD and 5 cm depth with the 9.96 × 9.96 cm^2^ field size. Film preparation, lecture and analysis were performed as described in Moignier et al. [26]. Four LiF µcubes and EBT3 films were used for each field size. Two series of measurements were carried out on two different days. Additional OF measurements at 80 cm SSD and 10 cm depth were performed with PTW 60019, PTW 60023 and SN Edge detectors for field sizes ranging from 0.415 × 0.42 cm^2^ to 9.96 × 9.96 cm^2^. For output factors with active detectors, prior CAX measurements allowed the effective point of measurement to be adjusted and the detector to be centered on the maximum peak of intensity. As active detectors response in small fields is perturbated due to volume averaging and lack of electronic equilibrium, the code of practice TRS 483 provides output correction factors (k$\frac{fclin, fmsr}{Qclin, Qmsr})$ to be used to correct output factors measurements in small radiation fields. For a better interpretation of the output factor results, k$\frac{fclin, fmsr}{Qclin, Qmsr}$ given by the IAEA TRS 483 and the ones from Weber et al*.* for the PTW 60023 [27] were applied to the values measured with the active detectors even if they are not published to correct measured OF under magnetic field. For the 1.66 × 1.66 cm^2^ field size, corrections factors were applied for PTW 31010, PTW, 31022, PTW 60019, PTW 60023 and SN edge detectors. For the 0.84 × 0.83 cm^2^ field size, only the three high resolution detectors output factors were corrected whereas for the smallest field size (0.42 × 0.415 cm^2^), values for the PTW 60019 only were corrected. Regarding the passive dosimeters, it has been shown that, on conventional linear accelerator, both don’t require correction factors for small fields [28, 29]. In addition, at magnetic field strength of 0.35 T, Darafsheh et al. [30] and Xhaferllari et al. [31] didn’t notice any significant difference in the response of EBT3 film irradiated in the presence or absence of an external magnetic field.

### Absolute dose verification for beam output calibration

To check the beam output calibration, dose point measurements were performed at 90 cm SAD and 1.5 cm depth (z_max_) for a 9.96 × 9.96 cm^2^ field size in a Beamscan MR water tank and in a Virtual Water phantom (Standard Imaging, WI, USA) with three ionization chambers (PTW 31010, PTW 31021 and Exradin A28 MR) suitable for reference dosimetry and positioned parallel to the magnetic field.

In addition, the consistency between calculated and measured dose with these three ionization chambers was investigated for beams of 9.96 × 9.96 cm^2^ field size, first in the daily phantom provided by Viewray with one beam at gantry 0°, secondly with the Delta 4 MR phantom (Scandidos, Uppsala, Sweden) with four beams at gantry 45, 110, 250 and 315° to avoid the two orthogonal detector plans. The use of different solid phantoms aimed at determining the less impacted ionization chamber by the air gap and therefore the most suitable for dose point measurement in different steps of the QA process.

The beam quality correction factors applied on the measurements for the absolute dose determination were the specific correction factors in presence of magnetic field k_B_,_Q,Q0//_ published by Krauss et al.

### Absolute dose verification for commissioning

#### Dose point measurements

Dose point measurements were performed using the most suitable ionization chamber determined in the previous section.

**Fig. 2:**
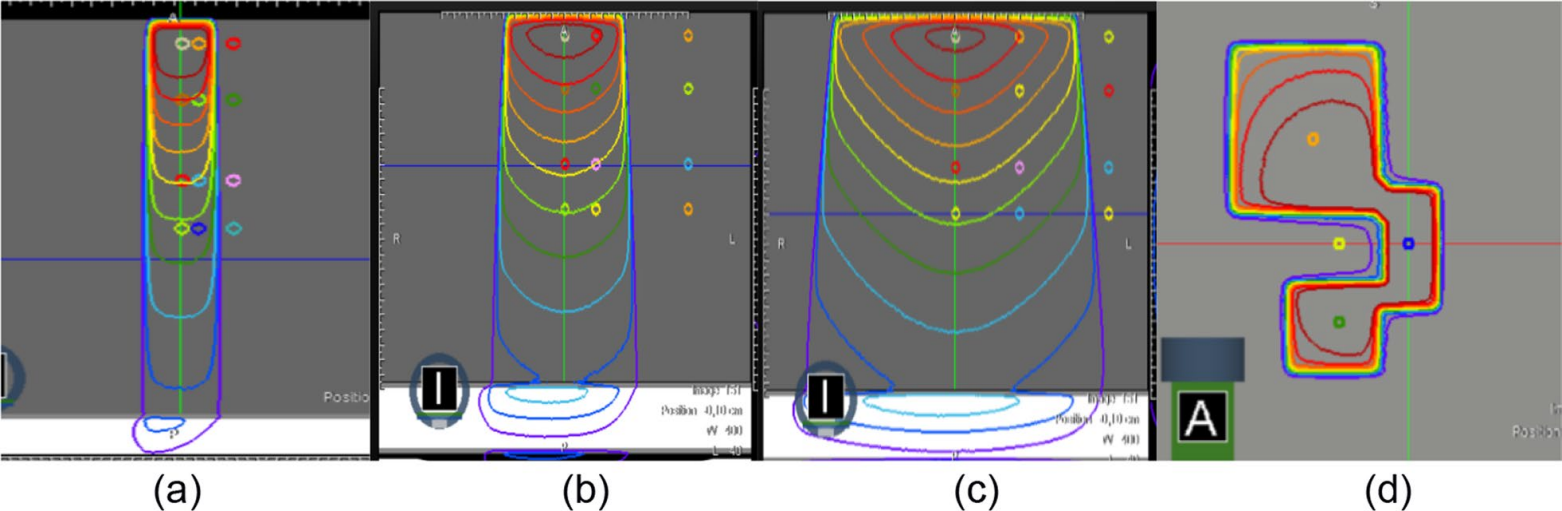
Beam specific calculation checks. Dose point verifications on the center axis, off-axis and out of the field for 3.32 × 3.32 cm^2^ (**a**), 9.96 × 9.96 cm^2^ (**b**), 19.92 × 19.92 cm^2^ (**c**) and a complex field (**d**)

The IAEA-TECDOC-1583 recommends performing some beam specific calculation checks for a small, a medium and a large field size [32]. Dose points verifications at SSD 78 and 85 cm were performed in the water tank for points located on the central axis, off-axis and out of the field at 1.5, 5, 10 and 13 (SSD 85 cm) or 20 (SSD 78 cm) cm depth for a 3.32 × 3.32, 9.96 × 9.96, 19.92 × 19.92 cm^2^ and a complex field size (Fig. 2).

Points in the field were assessed with relative error whereas points out of the beam used relative normalised error [32] (“Appendix B”).

#### End to end clinical tests

**Fig. 3:**
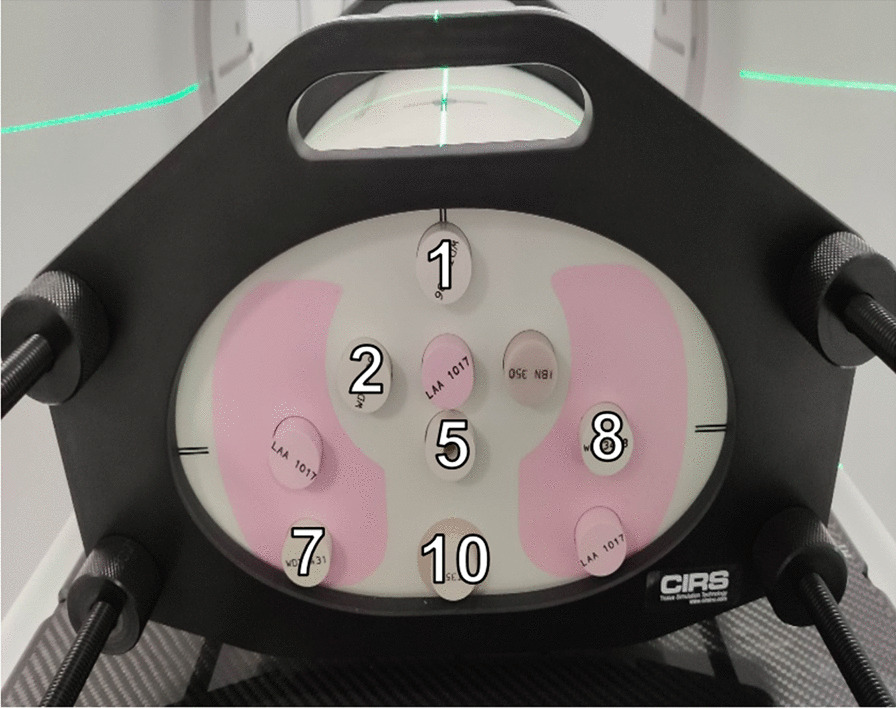
Dose points of the clinical test cases in the CIRS thorax phantom

The CIRS 002LFC thorax phantom (Sun Nuclear, FL, USA) was scanned on a Discovery RT Computed Tomography (GE, USA). On reconstructed CT images with a voxel size of 1 × 1 × 1.5 mm^3^, the clinical commissioning tests were done following the IAEA-TECDOC-1583 recommendations. The eight clinical cases were simulated on the TPS and the dose points reported were the mean dose in a 5 mm diameter sphere in the position 1, 2, 5, 7, 8 and 10 (Fig. 3).

Due to the specificity of the machine, no collimator rotations and no beam wedges were applied. The non-coplanar beam of case 8 was simulated with the Pseudo 4Pi function using the beam divergence. Each point was assessed with the relative reference normalised error [32].

#### Zeus algorithm validation

All the dose points measured during the two previous commissioning steps were used to validate Zeus, the secondary Monte Carlo calculation included in the Treatment Planning and Delivery Station (TPDS) and used for adaptive fractions as a secondary calculation check. Adaptive fractions were simulated on the MRIdian with the daily QA phantom that can provide enough MR signals. The original contours were rigidly copied and the original electronic density was applied. The 5 mm diameter sphere structures created in the original plan made it possible to have the mean Zeus calculated dose in the report.

### Statistical analysis

The smoothing and comparisons between the PDD, the dose profiles in X/Y directions as well as their associated parameters (penumbra, field size, unflatness and symmetry) were performed within the generalized additive models (GAM) framework [33].

More precisely, the measured PDD can be viewed as a noisy discretization of a two-dimensional continuous process modelled as a function of depth and field size. In the same way, the measured dose profile parameters can be presented as a noisy discretization of a one dimensional continuous process modelled as a function of field size. This function was estimated from the data as a summation of a smoothing (univariate or bivariate) spline function intercept representing Monte Carlo TPS calculations (the reference) and GAM interaction terms with each MR detectors.

**Fig. 4:**
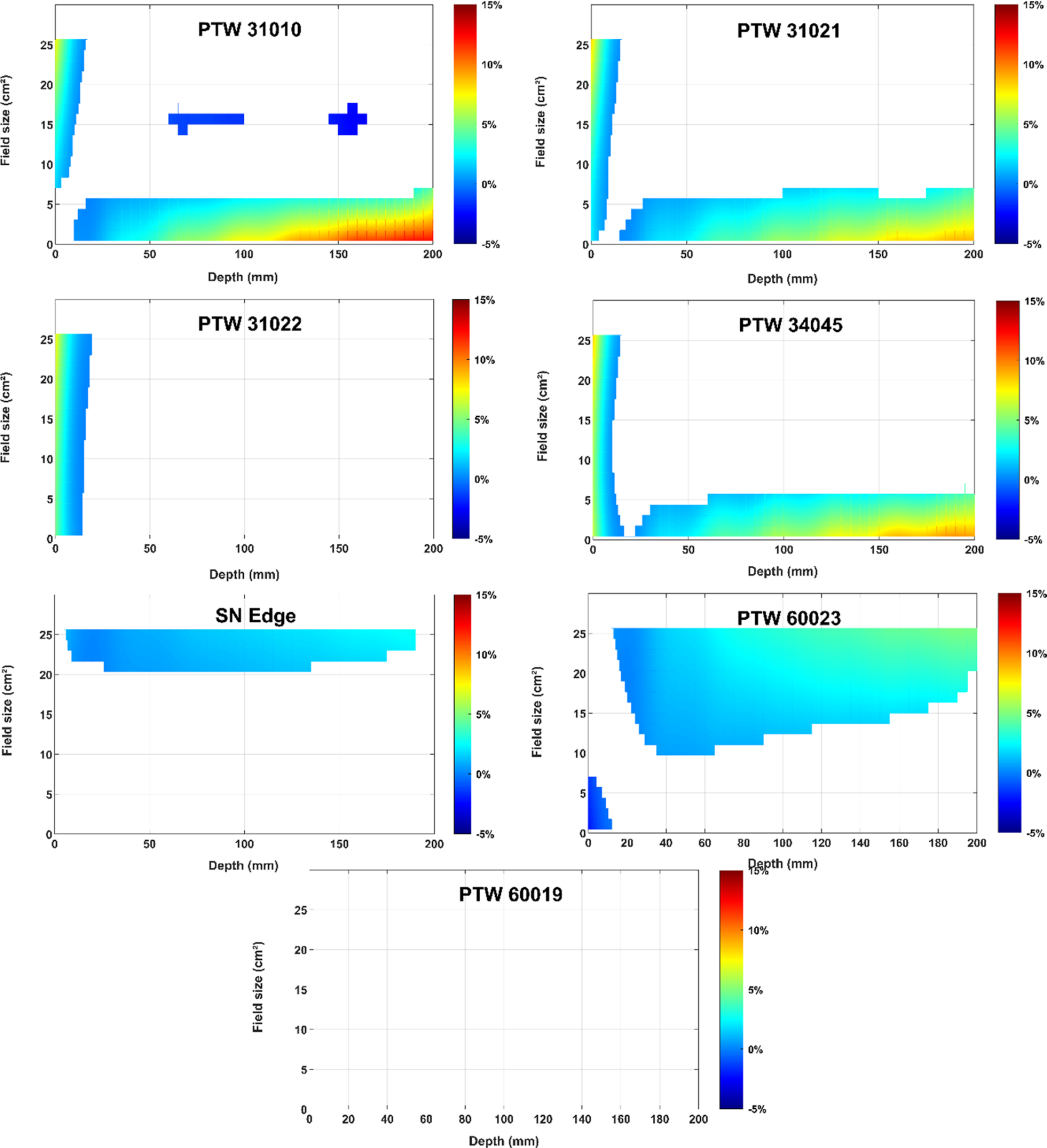
PDD absolute difference between TPS (reference) and the different detectors according to the field size and the depth. In white, deviations to TPS are not statistically significant

**Fig. 5:**
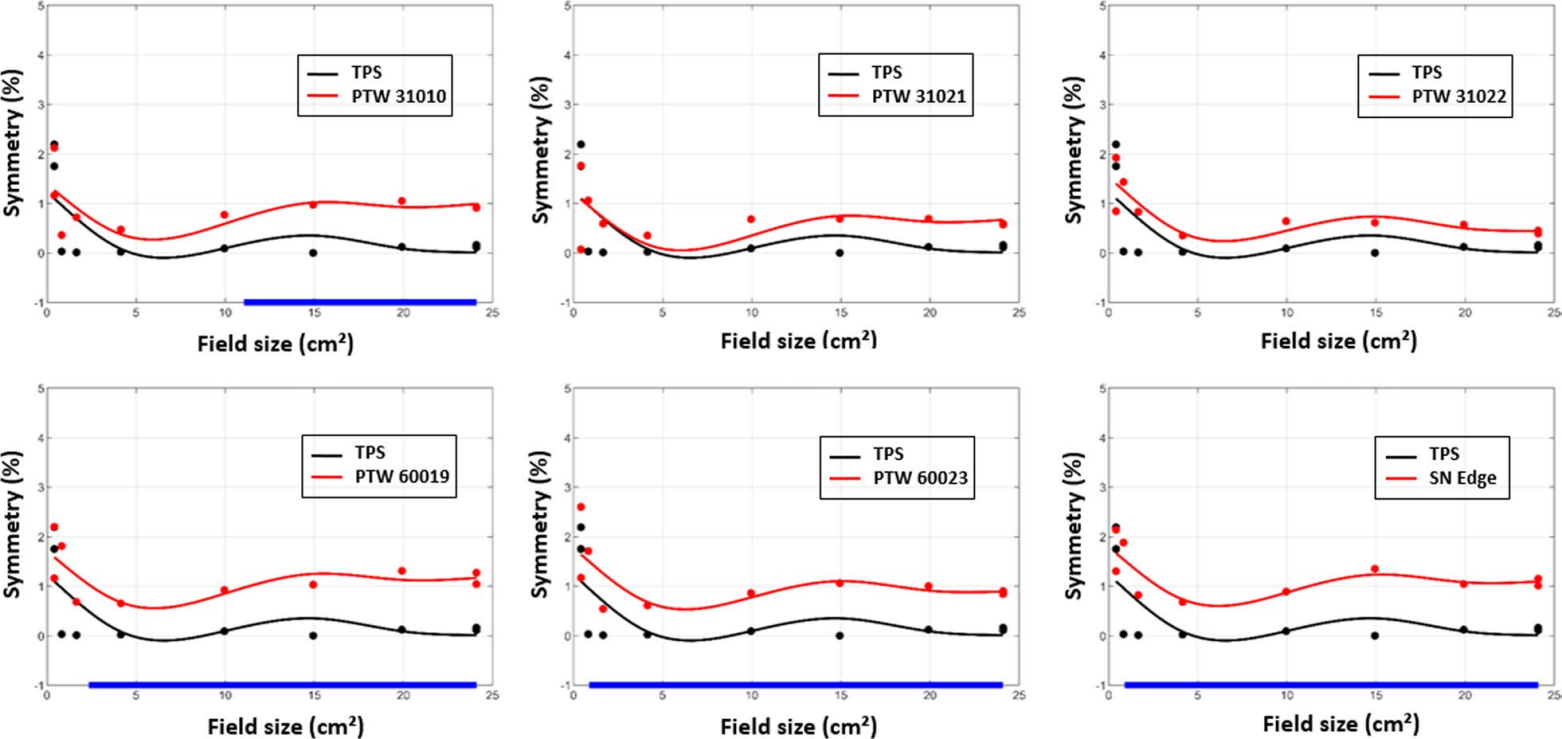
Y-profile symmetry of TPS data and detectors according to the field size with a multivariate regression approach. The blue segments in the x-axis highlight the range of values where the difference is statistically significant

**Fig. 6:**
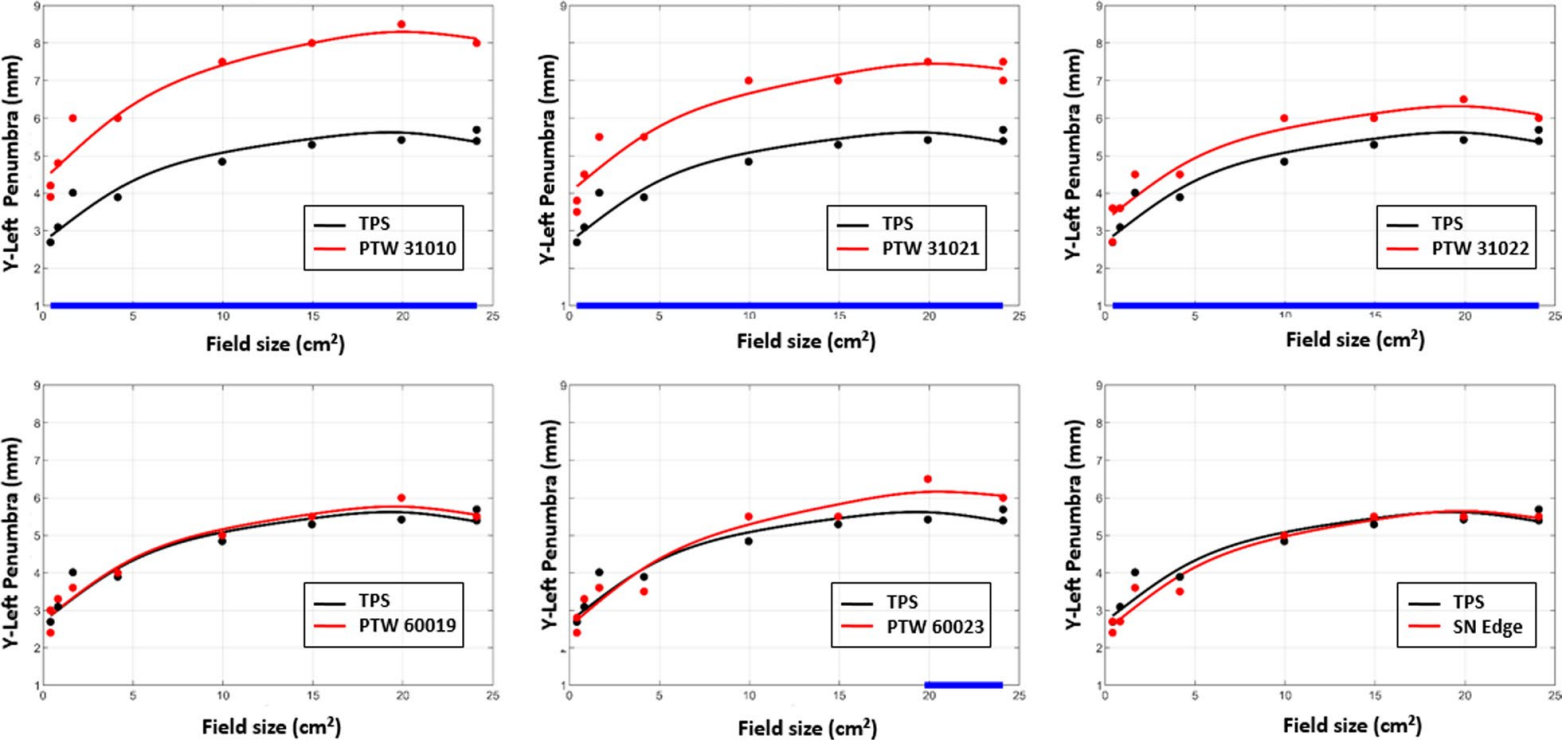
Y-profile left penumbra of TPS data and detectors according to the field size. The blue segments in the x-axis highlight the range of values where the difference is statistically significant

The comparisons between the TPS and the detector measurements was then based on the statistical inference of these interaction terms in the following way: the model provides pointwise estimation of the difference between TPS and the detector as well as the associated confidence intervals over all the continuum grid. Then, we considered in this study a significant difference when the confidence interval did not contain zero. This approach permitted to highlight the significant differences along the estimated curves and surfaces according to the field size and depth values (see Figs. 4, 5, 6, 8).

All analyses were performed using the mgcv package (https://cran.r-project.org/web/packages/mgcv/) of the R software version 4.2.1 2022.

## Main results

### Relative dose measurements for beam model validation

#### Percentage depth dose curves

The PDD absolute deviation to the TPS data along the depth and according to the field size for each detector included in this study are given in Fig. 4. The non-statistically significant differences appear in white.

In the build-up region, whatever the field size, the PDD measured with four ionisation chambers (PTW 31010, 31021, 31022 and 34045) were higher than those of the TPS, up to 7.5% for the PTW 34045. For the three largest ionization chambers (PTW 31010, 31021 and 34045), significant deviations were observed for field sizes under 5 × 5 cm^2^, up to + 12% with the PTW 31010 for the smallest field size at 200 mm depth. Except for the build-up region, the PTW 31022 measurements were in good agreement with the TPS data without significant differences whatever the field size.

PDD measured with the SN Edge and the PTW 60023 were higher than the reference over a wide depth range for field sizes larger than 20 × 20 cm^2^ and 10 × 10 cm^2^ respectively. The maximum deviation observed was + 5% for the PTW 60023 with the maximum field size (27.2 × 24.07 cm^2^) at 200 mm depth.

There were no statistically differences for the PTW microdiamond 60019 for the full range of field sizes.

#### Dose profiles

The GAM Analysis illustrates the evolution of measured and calculated profile parameters (penumbra, field size, unflatness and symmetry) as a function of field size using a multivariate regression approach. Regarding the four parameters investigated for the comparison of dose profiles, none of the detectors showed significant deviation from the TPS in terms of field size and unflatness for X and Y profiles. Therefore, only the most notable results are presented. The results obtained for the Y-profile symmetry, the Y-profile penumbra and the X-profile penumbra are presented in Figs. 5, 6 and 8 respectively. The blue segments on the x-axis highlight the range of values where the difference is statistically significant.

The symmetry for the three high resolution detectors exceeded the TPS data by around 1% in the Y direction whereas the three ionization chambers showed no significant differences overall except for the PTW 31010 for field sizes larger than 11 × 11 cm^2^ (Fig. 5). The symmetry in the X direction had the same trend.

Concerning the penumbras in the Y direction, the left penumbras measured by the high resolution detectors were in good agreement with the TPS values except for the PTW 60023 for large field sizes, the difference became significant of about + 0.5 mm beyond 20 × 20 cm^2^ whereas the ionization chambers showed a constant significant deviation of around + 0.7, 1.6 and 2.4 mm for the PTW 31022, 31021 and 31010 respectively (Fig. 6). The right penumbras followed the same trend in this direction.

**Fig. 7:**
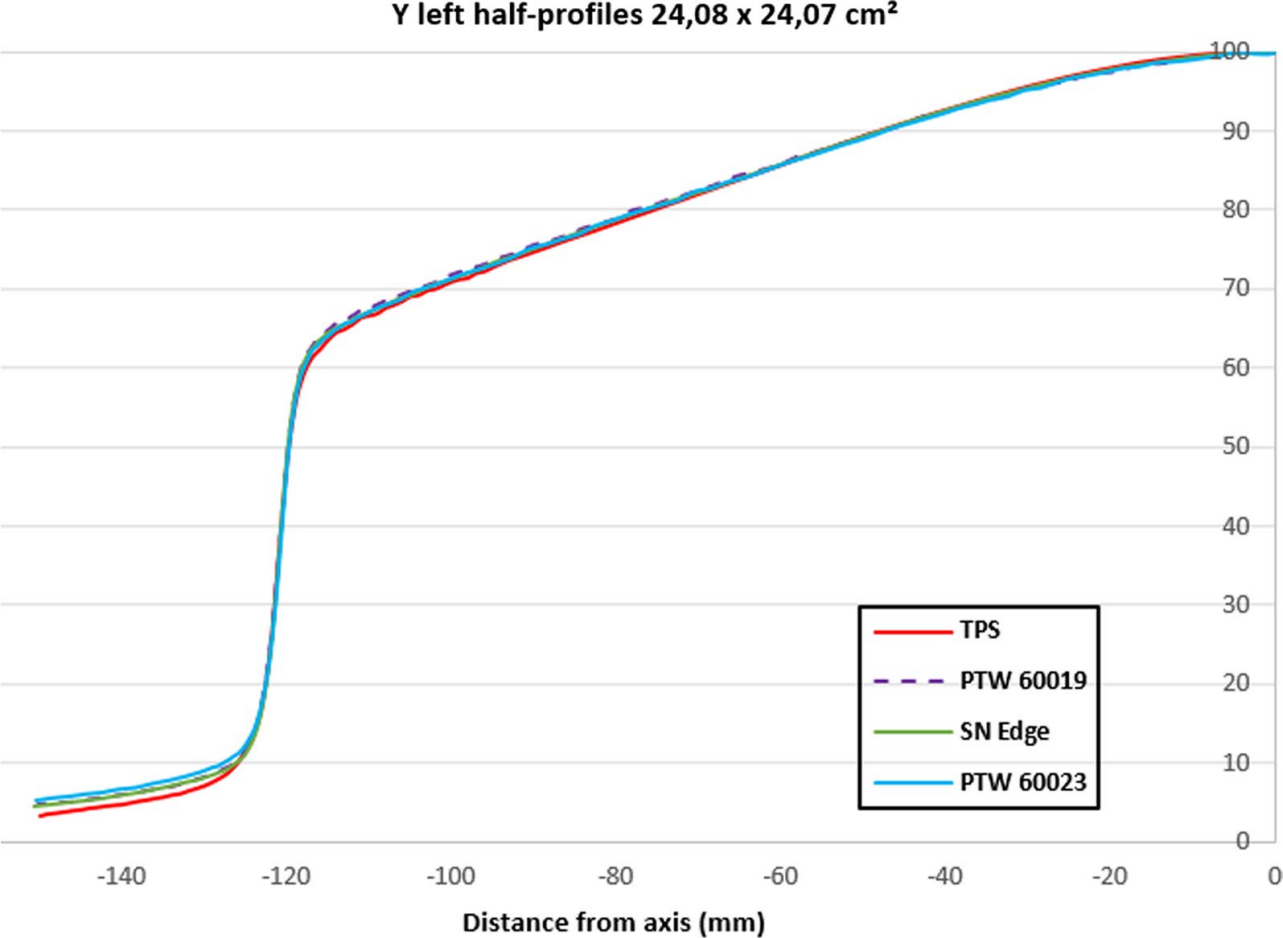
Y left half-profiles comparison between the TPS and the high resolution detector measurements for a 24.08 × 24.07 cm^2^ field size

The Fig. 7 is a focus on the Y left half-profiles for the 24.08 × 24.07 cm^2^ field size, the high resolution detectors were in good agreement with the TPS curve except out of the field where the PTW 60023 had the larger positive deviation to the TPS.

**Fig. 8:**
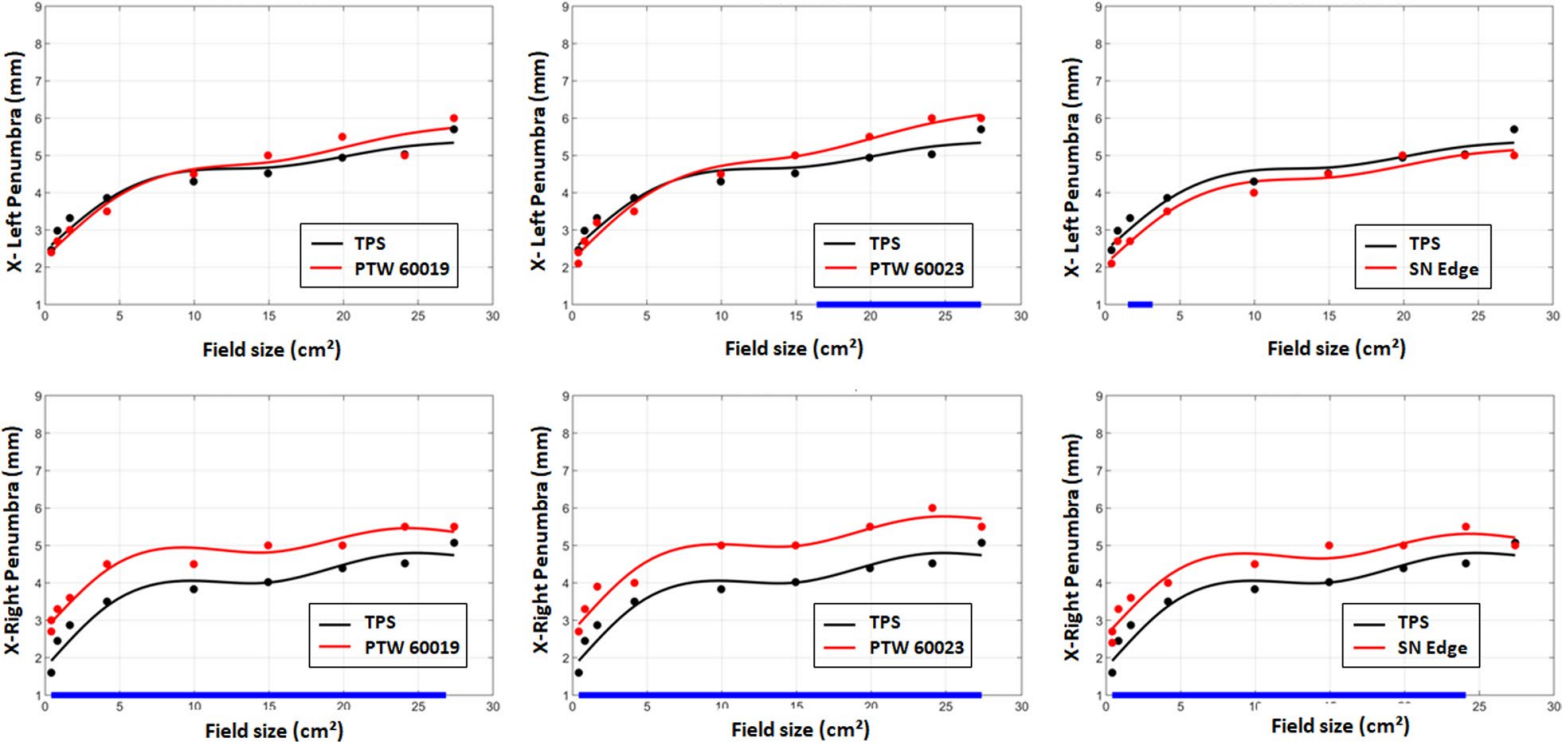
X-profile left and right penumbra of TPS data and high resolution detectors according to the field size. The blue segments in the x-axis highlight the range of values where the difference is statistically significant

For the left penumbras in the X direction, the PTW 60019 and SN Edge measured penumbras were in good agreement with TPS data and the PTW 60023 penumbras became significantly higher by around 0.5 mm beyond a 16 × 16 cm^2^ field size. The right penumbras measured by the three high resolution detectors were all significantly higher by around 1 mm than the TPS data (Fig. 8). The behaviour of the ionization chambers measured penumbras was the same for the right or left penumbras in the X or Y direction: significantly higher than TPS values with the smallest differences for PTW 31022 and the largest for PTW 31010.

**Fig. 9:**
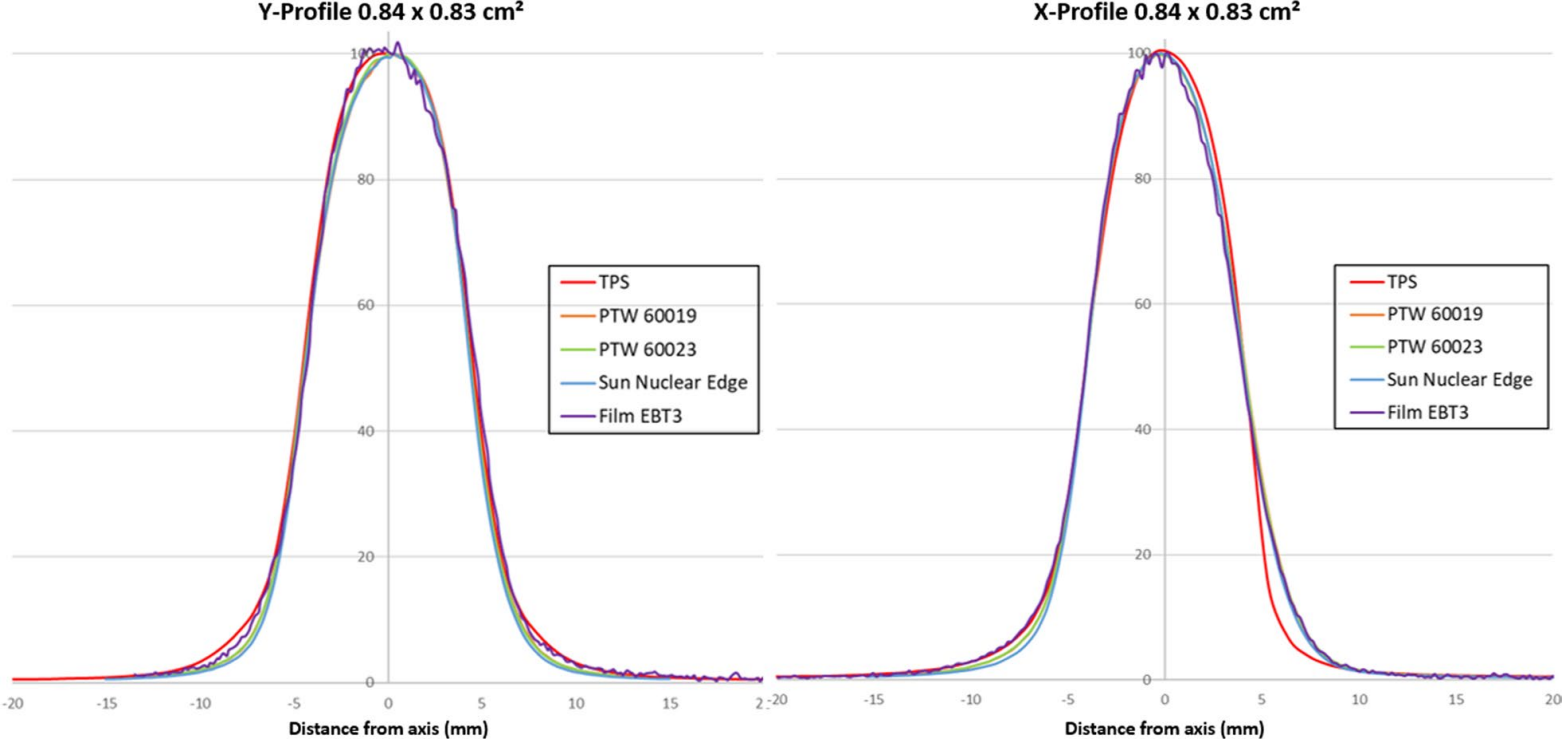
Profiles comparison (Y direction on the left graph and X direction on the right graph) between the TPS and the high resolution detectors and EBT3 measurements for a 0.84 × 0.83 cm^2^ field size

The Fig. 9 illustrates X and Y-Profiles comparison between the TPS and the detector measurements for a 0.84 × 0.83 cm^2^ field size. Additional EBT3 film profiles were added. The three high resolution detector X-profiles overlapped and differed from the TPS calculation whereas in the Y direction, all the high resolution measured profiles were in good agreement with the TPS profiles (Fig. 9). In contrast, in the X direction, the film profiles were in good agreement with the high resolution detectors profiles and not the TPS profiles.

#### Angular responses

**Fig. 10:**
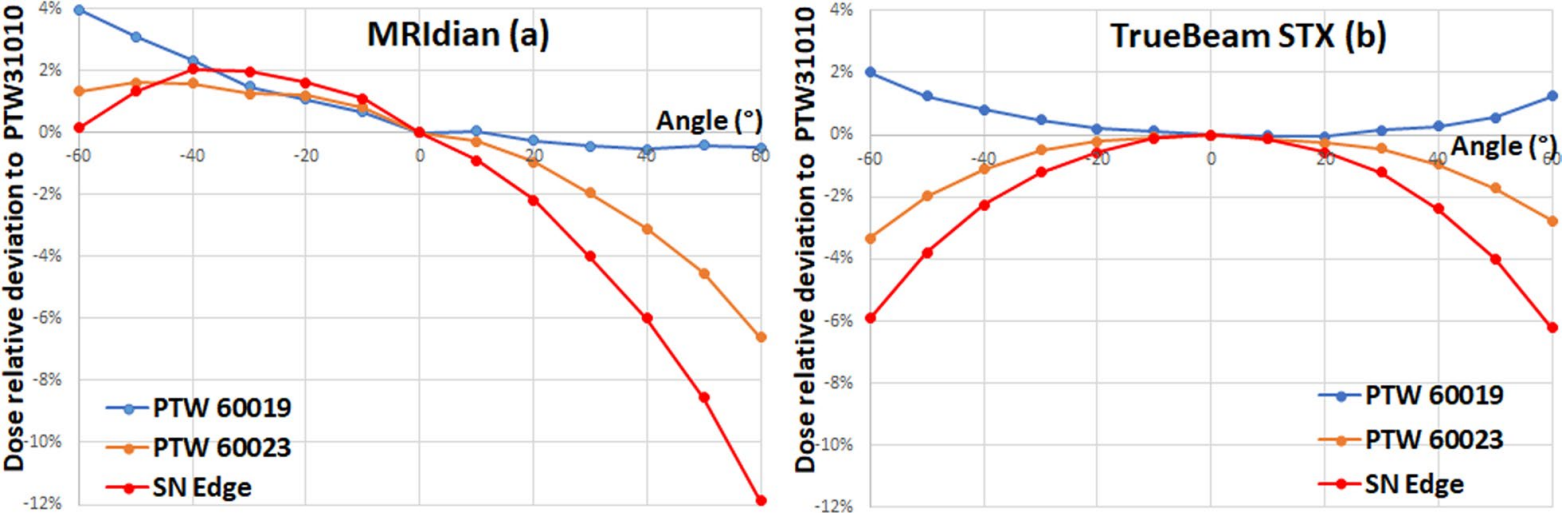
Angular response of high resolution detectors relative to PTW31010 ionization chamber at the MRIdian (**a**) and at the TrueBeam Stx (**b**)

At the MRIdian, the angular responses of the high resolution detectors were asymmetrical to 0° (Fig. 10a).

Compared to the PTW 31010, the detectors over-responded from − 60 to 0° whereas they under-responded at the opposite angles. The largest negative deviations observed were − 0.5, − 6.6 and − 11.9% at 60° for the PTW 60019, PTW 60023 and the SN Edge respectively. The largest positive deviations were + 1.6 and + 2% at − 40° for the PTW 60023 and the SN Edge whereas the PTW 60019 reached + 4% at − 60°.

At the TrueBeam Stx i.e. in absence of magnetic field, the angular responses were symmetrical to 0° (Fig. 10b). The PTW microdiamond over-responded with a 2% maximum positive deviation at − 60° whereas the PTW 60023 and SN Edge under-responded with a maximum negative deviation of 3.5% and 6% at − 60° respectively.

#### Output factors

**Fig. 11:**
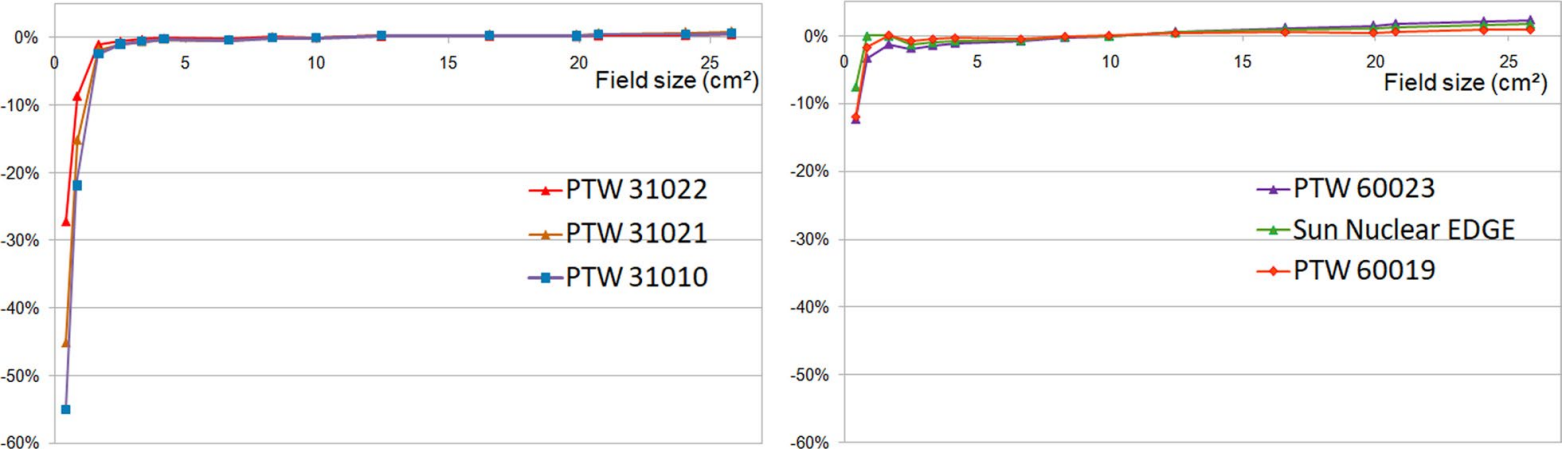
Output factors relative deviations to TPS for ionization chambers (left graph) and high resolution detectors (right graph)

Deviations between calculated OF and active detectors measured OF are presented in Fig. 11. Ionization chambers and PTW 60019 output factors were in good agreement with the TPS for large field sizes while OF underestimation is observed for the PTW 31010 and PTW 31021 from the 2.5 × 2.49 cm^2^ field size with a maximum deviation of − 55.1% and − 45.2% respectively for the smallest field size. The PTW 31022 and 60019 output factors relative deviations exceeded − 1% below the 1.66 × 1.66 cm^2^ field size and reached a maximum difference of − 27.3% and − 12% respectively for the 0.42 × 0.415 cm^2^ field size. A slight overestimation (1%) is observed for the diodes above the 16.6 × 16.6 cm^2^ field size and increased to 1.8% and 2.3% for SN Edge and PTW 60023 respectively at the largest field size. The SN Edge output factors relative deviation exceeded − 1% below the 0.84 × 0.83 cm^2^ field size and reached − 7.6% for the smallest field. Concerning the PTW 60023 results, differences above − 1% was found below 6.64 × 6.64 cm^2^ with a maximum deviation of − 12.3% for the 0.42 × 0.415 cm^2^ field size.

**Fig. 12:**
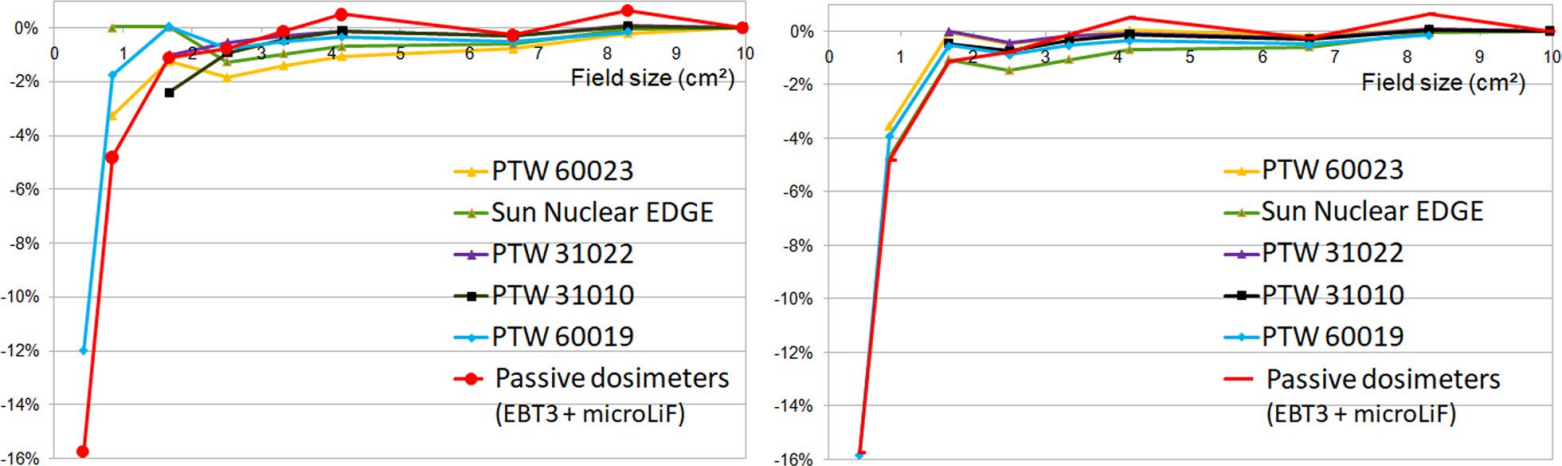
Small field output factors relative deviations to TPS for active and passive detectors (left graph). The results with* k*$\genfrac{}{}{0pt}{}{fclin,fmsr}{Qclin,Qmsr}$ corrected output factors are illustrated on the right graph

Figure 12 focuses on small field size output factor deviations for active and passive dosimeters for the three smallest field sizes. Only values with output correction factors k$\frac{fclin, fmsr}{Qclin, Qmsr}$ given by the IAEA TRS 483 and Weber et al. are presented. For the 0.42 × 0.415 cm^2^ field size, the deviation between the PTW 60019 OF and the TPS OF went from − 12% before correction to − 15.9% after correction and matched the passive dosimeters deviation (− 15.7 ± 0.2%). For the 0.84 × 0.83 cm^2^ field size, the mean deviation from TPS for the three high-resolution detectors was − 1.7 ± 1.6% for uncorrected values versus − 4.0 ± 0.6% for corrected values, while a deviation of − 4.8 ± 0.8% was obtained for passive dosimeters compared to the TPS. For the 1.66 × 1.66 cm^2^ field size, the mean deviation to the TPS for uncorrected and corrected values of the five detectors went from − 0.9 ± 1% to − 0.4 ± 0.4% respectively whereas the passive detectors had a mean deviation of − 1.1 ± 1.5%.

Additional OF measurements performed with PTW 60019, PTW 60023 and SN Edge detectors at SSD 80 cm and 10 cm depth are presented in Table 2. As the field size decreases, the relative standard deviation of the output factors measured with the three combined detectors increases by 2% for the 0.84 × 0.83 cm^2^ field size. After correction with the k$\frac{fclin, fmsr}{Qclin, Qmsr}$ factors, all relative standard deviations fell below 0.5% regardless of field size. The 0.42 × 0.415 cm^2^ output factor was not reported because only the PTW 60019 was corrected and no relative standard deviation could be calculated.

**Table 2:** Additional output factors at SSD 80 cm, 10 cm depth

Field size (cm^2^)	Mean measured OF	Relative standard deviation (%)	Mean corrected OF	Relative standard deviation
0.84 × 0.83	0.643	2.0	0.628	0.5
1.66 × 1.66	0.787	1.0	0.785	0.4
2.5 × 2.49	0.828	0.8	0.831	0.4
3.32 × 3.32	0.858	0.7	0.861	0.4
4.16 × 4.15	0.885	0.6	0.888	0.3
6.64 × 6.64	0.945	0.3	0.947	0.2
8.3 × 8.3	0.975	0.2	0.976	0.1
9.96 × 9.96	1.000	0.0	1.000	0.0

Mean output factors measured with PTW 60019, PTW 60023 and SN Edge as well as the mean values of *k*$\genfrac{}{}{0pt}{}{fclin,fmsr}{Qclin,Qmsr}$ corrected output factors are reported with relative standard deviations

### Absolute dose verification for output calibration

The mean absolute dose deviations to the TPS in different phantoms were 0 ± 0.4%, − 1.2 ± 0.6% and 0.5 ± 1.1% for the PTW 31010, PTW 31021 and Exradin A28MR chambers respectively.

### Absolute dose verification for commissioning

Given the results obtained for output calibration, all the measurements were acquired with the PTW 31010.

#### Dose point measurements

The relative and relative normalised errors obtained for the 104 points investigated were below 3%. The mean deviation was − 0.4 ± 0.9% and the maximum error was − 2.8% for the off-axis point of the 3.32 × 3.32 cm^2^ field size at 20 cm depth.

#### End to end clinical tests

The results obtained for 8 clinical cases are reported in Table 3. All relative reference normalised errors were below 2% and met the IAEA-TECDOC-1583 criteria. The mean deviation was − 0.2 ± 0.8% and the maximum error was − 1.7% for point 5 of case n°6.

**Table 3:** Relative reference normalised errors between the ionization measurements (PTW 31010) and the TPS calculated dose in the CIRS 002 LFC thorax phantom for 8 clinical cases

Clinical case	No. 1	No. 2	No. 3	No. 4	No. 5	No. 6	No. 7	No. 8
*Relative reference normalised error (%)*								
Point 1	0.9%	− 0.4%						
Point 2					0.1%			
Point 5	0.3%		0.2%	− 0.1%		− 1.7%	− 0.3%	− 1.0%
Point 7	0.3%			0.4%	− 1.7%	− 1.3%		
Point 8				− 0.3%				
Point 10	− 0.5%			0.8%		0.9%		

#### Zeus algorithm validation

The relative difference between the measured dose points in the water tank and Zeus calculation was − 0.4 ± 1.4% with a maximum deviation of − 3.5% for the off-axis point in the 3.32 × 3.32 cm^2^ field size at 20 cm depth. The deviation for the clinical cases was − 0.4 ± 1.1% with a maximum at − 2.3% for point 5 of case n°6.

The mean relative deviation between Zeus calculation and the TPS Monte Carlo calculation was 0.3 ± 0.6% with a maximum at 1.8% for the off-axis point in the 3.32 × 3.32 cm^2^ field size at 10 cm depth.

## Discussions

For PDD measurements, no significant difference was observed between the detectors measurements and the TPS Monte Carlo calculations for the range of field sizes recommended by the manufacturer. An excellent agreement was obtained with the microdiamond PTW 60019 over the full range of field sizes. For PDD, field size varies with depth so the relative response of a detector may differ according to the size of its sensitive volume. Consequently, it is likely that the over-response of PTW 31010 and 31021 ionization chambers, for field sizes below 5 × 5 cm^2^, is due to an under-estimation of the dose at the depth of maximum dose because of the volume averaging effect. The larger the active volume (PTW31010), the higher the over-response. For the ionization chamber with the smallest active volume (PTW31022) a good agreement with TPS was observed even for very small field sizes. Regarding the over-response of diodes for large field sizes, it is due to the increase of scattered photons in large field sizes and at larger depths for which the diode over-responds because of the silicon mass absorption coefficient energy dependence. The SN Edge which is shielded has a lower over-response than the unshielded PTW60023.

The angular dependences reported in Fig. 10 attests the non-negligible impact of the magnetic field even at this low level of intensity. The comparison between the MRidian and the Truebeam STx affirms clearly that the asymmetrical angular is induced by the presence of the magnetic field. In presence of magnetic field, the results agreed with the microdiamond angular response reported by Woodings et al. [15]. Indeed, in our study, the magnetic field B0 was lower and pointed in the opposite direction. As a result, the over-response was weaker and the trend of the curve was reversed around 0°. In agreement with angular responses reported for the high resolution detectors, ionization chambers were more suitable to evaluate the profile symmetry. In contrast, their collecting volume was a drawback for the penumbra assessments. For the unflatness and symmetry assessments, the PTW 310221 and 31022 ionization chambers showed the best results.

In the Y direction, where the magnetic field does not influence the profile, there were no statistically significant differences in the penumbra assessment for high resolution detectors except for PTW 60023 at large field sizes. Indeed, for the latter, the out of the field over-response disturbed and slightly overestimated the reported penumbra (Fig. 7).

The over-response due to the silicon mass absorption coefficient energy dependence increased with the field size because of the increasing proportion of scattered photons. Figures 7 and 9 clearly illustrate the out of the beam silicon over-response for a large field.

In the X direction, for left penumbras, we found the same significant results than the Y direction confirming the overestimation of PTW 60023 due to the over-response out of the large fields. However, even if the differences were not statistically significant, the left penumbras measured with the SN Edge was the only case where the measured penumbras were lower than the TPS values. This last trend is probably attributable to SN Edge angular response with the strong under-response measured from 0 to 60°. Given that the magnetic field B0 is pointing into the bore, the secondary electrons are deviated following the Lorentz force in a circular trajectory towards the patient’s right in a head first supine position (Linac left). In the left penumbra region, scattered electrons subjected to the Lorentz force can reach the detector from various positive angles. For the right penumbras, high resolution detectors found significant higher values. The angular dependences from 0 to − 60° were too weak to explain the right penumbras deviations. Moreover, on this side of the field, the circular trajectory direction in the water reduced the angle spread of the electrons reaching the detectors. To clarify the angular response contribution, EBT3 film profiles were added in the X and Y profile graphical comparison for a 0.84 × 0.83 cm^2^ field size (Fig. 9). EBT3 films response are known to be angular independent [34] and not sensible to 0.35 T magnetic field exposure [31]. In the Y direction, all the measured profiles including the films matched the TPS curve. In contrast, in the X direction, the EBT3 film profile confirmed the high resolution detector measurements which were at odds with the TPS. In comparison, TPS right and left penumbras were slightly asymmetric whereas all the measured penumbras were rather symmetric. Based on these observations, we can assume that the Monte Carlo calculations of this version of TPS slightly overestimate the effects of the magnetic field.

Under a 1.5 T magnetic field, O’Brien et al. [13] found that the shielded diodes hid the magnetic field effect on the dose profiles. In our study, at low level of magnetic field, the behaviour of the shielded diode was similar to the unshielded diode and the microdiamond. The profiles visually overlapped and showed an equivalent unflatness, symmetry and penumbras. Only the left penumbras were slightly steeper probably because of the angular response. For the penumbra measurement, the PTW60019 microdiamond represented the best compromise between the collecting volume, the angular response and the out of the field over-response.

The ionization chambers and the TPS output factors were in good agreement as long as they were not affected by the volume averaging effect. When the field size decreased, the deviations increased with the sensitive volume. The PTW 31022 was the most suitable ionization chamber. As for the PDD, shielded and unshielded diodes over-responded at large field size because of the silicon mass absorption coefficient energy dependence. PTW 60019 was the detector that showed the less deviation to the TPS values for the full range of field sizes. Valdenaire et al. [19] also found an agreement between the PTW 60019 and the TPS OF within 0.8% for field sizes down to 1.66 × 1.66 cm^2^.

When the output correction factors k$\frac{fclin, fmsr}{Qclin, Qmsr}$ were applied, the spread of the corrected values was considerably reduced for the smallest field sizes at 5 cm and 10 cm depth. Moreover, at 0.35 T, Blum et al. [14] found small field output correction factors close to the free magnetic field values for the PTW 60019 and 60023. That’s why, even though the output correction factors were published for measurements without magnetic field at 10 cm depth, they seem to be applicable at low magnetic field at 5 cm depth pending the publication of new MR specific values. The mean corrected values deviated from the TPS output factors and converged to the passive dosimeters output factors. Based on these observations, we can question the Monte Carlo output factor calculations of this version of TPS for very small field sizes and have more confidence in the passive dosimeter values. Lim et al. [17] found a similar PTW 60019 response compared with films under a 1.5 T magnetic field. The PTW 60019 corrected output factor was + 1.2% higher for a 1 × 1 cm^2^ field size whereas we observed + 0.9% for a 0.84 × 0.83 cm^2^ field size in our study.

PTW 31010 ionization chamber matched Monte Carlo absolute doses with high accuracy in the different materials and was the most suitable detector for absolute dosimetry. This latter was chosen as reference detector for all the commissioning tests requiring absolute dose verification. These results were valid for the oriented detector parallel to the magnetic field accordingly to Margaroni et al. [9] who concluded that effects related to air gap, field size, depth and material were minimal in this orientation under a 1.5 T magnetic field. In our study, according to the different phantoms used, the air gap varied from 0.1 to 0.9 mm so at low magnetic field, the PTW well-oriented 31010 showed negligible air gap effect. The PTW 31021 under-responded of 1.2% despite a calibration at the same date in the same dosimetry laboratory. The size of the active volume could explain this difference. As a matter of a fact, two studies [8, 9] with various ionization chamber sensitive volumes reported different results of the asymmetrical air gap effect at 1.5 T. Compared without air gap, O’Brien et al. [8] had a decreased dose of 1.6% in the active volume for a 0.2 mm asymmetrical air gap with a Farmer type chamber whereas Margaroni et al. [9] had − 3% in a PTW 31021 small-cavity chamber. The Exradin A28 MR, recommended by Viewray for the system acceptance tests (SAT) had the largest standard deviation. All the commissioning dosimetric tests respected the IAEA-1583 TECDOC criteria with excellent results especially in heterogeneous conditions even if the CIRS Thorax phantom had additional lung and bone inserts. Thanks to all the measured points, the secondary Monte Carlo Zeus could have been validated and also compared to the primary TPS Monte Carlo calculation with a good agreement.

## Conclusions

Compared to the literature at 1.5 T, the magnetic field effects on the measurements are considerably reduced at low magnetic field. The PTW 31010 ionisation chamber, when well oriented, can be used with confidence in a variety of materials and phantoms for commissioning and QA tests requiring absolute dose verifications. For relative measurements, all detectors were in good agreement with TPS calculations as long as they were employed in their range of use. The PTW 60019 showed the best agreement across the whole range of field sizes for the PDD and the output factors. Ionisation chambers were limited by their active volume but were the most suitable detectors for the symmetry assessment. Shielded and unshielded diodes over-responded at large field sizes due to the silicon energy dependence. At this level of magnetic field, shielded diodes have a similar behaviour to the PTW 60019 and 60023 for the profile assessment in terms of unflatness, symmetry and penumbras. Only PTW 60023 showed a slight overestimation of the penumbra for large field sizes because of the out-of-field over-response. For the penumbra measurement, the PTW60019 microdiamond represented the best compromise between the collecting volume, the angular response and the out of the field over-response. The output correction factors published by the IAEA TRS 483 seem to be applicable at low magnetic field pending the publication of new MR specific values. The spread of corrected OF were considerably reduced and converged to the passive dosimeters OF.

## Data Availability

The datasets supporting the conclusions of this article are included within the article.

## Appendix A: Definition of the dose profile parameters

The field size was determined by the distance between the inflexion points [24].

The penumbra was the distance between the 40% and the 160% of the isodose level at the inflexion point [24].

The unflatness for flattening filter free (FFF) beams was defined as [25]:

$$Unflatness = \frac{Dose\;on\;axis}{{Dose\;off\;axis }}$$

The dose off axis was at 60 and 80% of the field size for nominal field size < 10 cm and ≥ 10 cm respectively.

The symmetry was defined as an area ratio [25]:

$$Symmetry = \frac{{\left| {Left\;Integral - Right\;Integral} \right|}}{{\left| {Left\;Integral + Right\;Integral} \right|}} \times 2$$

where Integral was the area bounded by the central axis and the inflexion point.

## Appendix B: Evaluation of the absolute dose measurements for commissioning (“Absolute dose verification for commissioning” section)

### Evaluation of the dose point measurements (“Dose point measurements” section)

The relative error was defined as:

$${\text{Relative}}\;{\text{error}}\left( \% \right) = {1}00 \times \frac{{\left( {Dmeas - Dcalc} \right)}}{Dcalc}$$

where D_cal_ was the calculated dose by the TPS and D_meas_ was the measured dose with the detector.

The relative normalised error is related to dose on axis at the same depth and defined as:

$${\text{Relative}}\;{\text{normalised}}\;{\text{error}}\left( \% \right) = 100 \times \frac{{\left( {Dmeas - Dcal} \right)}}{Dcal,cax}$$

where D_cal,cax_ was the TPS calculated dose on the central axis at the same depth.

### Evaluation of the measurements of the end to end clinical tests (“End to end clinical tests” section)

Each point was assessed with the relative reference normalised error which was defined as [32]:

$${\text{Relative}}\;{\text{reference}}\;{\text{normalised}}\;{\text{error}}\left( \% \right) = 100 \times \frac{{\left( {Dmeas - Dcal} \right)}}{Dcal,ref}$$

where D_cal,ref_ was the TPS calculated dose at the reference point of each clinical case.

## Abbreviations

CAX: Central axis
CT: Computed tomography
IMRT: Intensity modulated radiation therapy
FFF: Flattening filter free
FS: Field size
MC: Monte Carlo
MLC: Multi leaf collimator
MRI: Magnetic resonance imaging
OF: Output factors
PDD: Percentage depth dose
QA: Quality assurance
SAD: Source axis distance
SSD: Source skin distance
TPS: Treatment planning system
VMC: Voxel Monte Carlo
